# Mannose-Binding Lectin: Biologic Characteristics and Role in the Susceptibility to Infections and Ischemia-Reperfusion Related Injury in Critically Ill Neonates

**DOI:** 10.1155/2017/7045630

**Published:** 2017-01-26

**Authors:** Cinzia Auriti, Giusi Prencipe, Maria Moriondo, Iliana Bersani, Chiara Bertaina, Vito Mondì, Rita Inglese

**Affiliations:** ^1^Department of Medical and Surgical Neonatology, Bambino Gesù Children's Hospital (IRCCS), Piazza S. Onofrio 4, 00165 Rome, Italy; ^2^Department of Laboratories, Laboratory of Rheumatology, Bambino Gesù Children's Hospital (IRCCS), Piazza S. Onofrio 4, 00165 Rome, Italy; ^3^Department of Pediatrics, Anna Meyer Children's University Hospital, Viale Gaetano Pieraccini 24, 50139 Florence, Italy; ^4^Department of Laboratories, Laboratory of Chemical Chemistry, Bambino Gesù Children's Hospital (IRCCS), Piazza S. Onofrio 4, 00165 Rome, Italy

## Abstract

The mannose-binding lectin (MBL) is a member of the collectin family, belonging to the innate immunity system. Genetic, biologic, and clinical properties of MBL have been widely investigated throughout the last decades, although some interesting aspects of its potential clinical relevance are still poorly understood. Low circulating concentrations of MBL have been associated with increased risk of infection and poor neurologic outcome in neonates. On the other hand, an excessive and uncontrolled inflammatory response by the neonatal intestine after the exposure to luminal bacteria, leading to an increased production of MBL, may be involved in the onset of necrotizing enterocolitis. The purpose of the present review is to summarize the current knowledge about genetic and biologic characteristics of MBL and its role in the susceptibility to infections and to ischemia-reperfusion related tissue injuries to better explore its clinical relevance during the perinatal period and the possible future therapeutic applications.

## 1. Introduction

The mannose-binding lectin (MBL) is a protein of the innate immune system, belonging to the collectin family, able to deploy a variety of antimicrobial activities. It recognizes and binds various pathogens (including bacteria, viruses, fungi, and parasites), providing protection against the microbial invasion of the host [1]. Although the clinical impact of MBL deficiency and its association to a wide variety of diseases has been extensively studied, the clinical significance of low MBL serum levels in healthy subjects is still debated. The image is that of a mosaic, as studies suggest a detrimental or beneficial or no impact of low or high MBL serum levels on the susceptibility to different diseases. In early life MBL insufficiency seems to have clinical relevance in the presence of immunodeficiency and whenever the immune system is particularly challenged [2]. Consecutively, MBL could play a critical role in the first line defence during the neonatal period, when the maternal-derived antibodies disappear and the child's own immune system is immature [3, 4]. In the same period of life, MBL seems also to play a role in contact guidance of neuronal migration, interneuronal recognition, myelinization, and tightening of the ependymal cell barrier [5].

While low MBL serum levels have been associated to an increased risk of nosocomial sepsis [6, 7] and of neurological risks [8] in neonates, recent studies performed in rodents support the role of MBL in the exacerbation of tissue damage (myocardial, gastrointestinal, cerebral, and renal tissues) in the course of ischemia-reperfusion injuries, by the activation of the lectin pathway of the complement. According to these data, we found that MBL-2 genotypes associated with high MBL serum levels represent a risk factor for necrotizing enterocolitis (NEC) in preterm neonates [9].

In conclusion the role of MBL, the exact clinical significance of the different MBL haplotypes and, consecutively, the associated serum MBL levels, is still poorly understood and needs to be further elucidated. Furthermore, it is still unclear if the exogenous administration of MBL may have protective or rather harmful effects on the host's organism [10].

The purpose of the present review is to summarize the current knowledge on the clinical role of MBL, especially during the perinatal period, and address controversial issues, discussing at the end on its possible future therapeutic applications.

## 2. MBL: Protein and Biologic Properties

**Figure 1:**
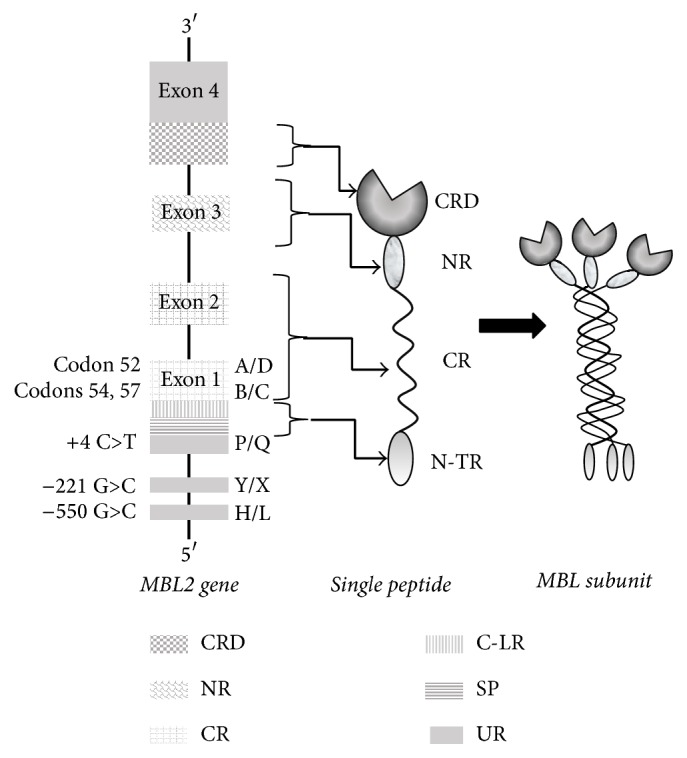
The MBL gene and the MBL structure. The organization of the* MBL* gene is located at chromosome 10q21. Two promotor polymorphisms at positions −550 and −221 are indicated. A third polymorphism is found at position +4. Exon 1 of the gene encodes the untranslated region (UR), the signal peptide (SP), and the cross-linking region (C-LR) of the N-terminal and the first part of the collagenous region (CR) harbouring the base mutations that results in the production of the MBL variants. The second exon encodes the remaining part of the collagenous region (CR) including the disruption of the Gly-X-Y repeat. A third exon encodes the neck region (NR). The last exon encodes the carbohydrate-recognition domain (CRD). The MBL subunit is composed of three single peptides.

The human* MBL2* gene product is a 24 kD polypeptide characterized by a 248-amino-acid sequence, with four distinct regions, a cysteine-rich N-terminal region, a collagenous domain, a short *α*-helical coiled-coil domain, the so-called neck region, and a carbohydrate-recognition domain, and forms the prominent globular head of the molecule. Three polypeptide chains form a triple helix through the collagenous region, stabilized by hydrophobic interaction and interchain disulphide bonds within the N-terminal cysteine-rich region. This trimeric form is the basic structural subunit of all circulating forms of MBL. Larger molecules can be obtained by the oligomerization of these homotrimeric subunits [11, 12] (Figure 1). The highly ordered oligomeric structure, the spacing, and orientation of the carbohydrate-recognition domains define what ligands MBL can target and are essential for its function. Through the carbohydrate-recognition domain MBL binds to specific carbohydrates such as mannose or N-acetylglucosamine that are exposed on the surface of a number of pathogens such as bacteria, viruses, parasites, and fungi [13–15]. For this reason, MBL belongs to the group of the so-called “pattern recognition molecules” [16] that mediate the precocious activation of the immune response. MBL is produced by the liver [15, 17–19] and released in the serum under stress conditions [20, 21] as a calcium-dependent acute phase protein. Significantly increased circulating levels have been reported in response to infections. During inflammatory conditions MBL can also leave the blood stream due to vascular leakage and can be detected in the mucus of the middle ear, in upper airway secretions, in inflamed synovial fluid, and in the normal amnion fluid [22, 23]. MBL activates macrophages [24], enhances phagocytosis [25, 26], and plays a role in complement activation by inducing the antibody-independent lectin pathway [1, 13, 16, 27–31]. In particular, MBL, in cooperation with three MBL-associated serine proteases (MASPs 1, 2, and 3), is able to initiate the lectin pathway of complement activation, the release of cytokines, and coagulation factors. A single MASP entity was initially identified and characterized as a protease with the ability to cleave complement proteins C4, C2, and C3 [31, 32]. MASP was indeed a mixture of two related but distinct proteases, MASP-1 and MASP-2 [30]. A third protease, MASP-3, is also shown to be associated with MBL [33]. It is generally believed that MASP-2 is the initiator of the lectin-complement pathway, while the role of the other MASPs is still uncertain [34]. MASPs additionally form active complexes with Ficolin-1 (M-Ficolin), Ficolin-2 (L-Ficolin), and Ficolin-3 (H-Ficolin), which are also defence collagens [35–37].

In the case of tissue damage after ischemia-reperfusion, MBL rapidly deposits on target cells and forms an IgM-MBL complex as soon as a specific autoreactive IgM binds to exposed tissue antigens and triggers the downstream complement activation in the acute phase, enhancing the cleavage of C3 [38]. Small amounts of MBL are also produced in organs other than the liver such as brain [39], kidney [40, 41], spleen [42], tonsil [43], thymus, small intestine [44], testis [42, 44], ovary [41], and vagina [45], suggesting that local expression of MBL may be relevant in local immune defence.

## 3. MBL: Genetics

In 1989, the gene structure of MBL and the protein were identified by Taylor and Sastry [48, 49]. The human MBL gene (*MBL2)* was cloned and sequenced and has been located in the chromosome 10q11.1–q21. Comparison of the genomic nucleotide sequence of* MBL2* with the cDNA sequence revealed that the protein-coding region consists of four exons interrupted by three introns of 600, 1350, and 800 base pairs in size, respectively. Exon 1 encodes the signal peptide, a cysteine-rich domain, and seven copies of a repeated glycine-Xaa-Yaa motif typical for the triple helix formation of collagen structures (Xaa and Yaa indicate any amino acid). This pattern is continued by additional 12 glycine-Xaa-Yaa repeats in exon 2. Exon 3 encodes a neck region and exon 4 a carbohydrate-binding domain. The resulting protein consists of oligomers each with three identical polypeptide chains of 32 kDa as evaluated on Sodium Dodecyl Sulfate Polyacrylamide Gel Electrophoresis (SDS-PAGE). The liver synthesizes the protein as structures consisting of three–six oligomers [50] (Figure 1).

### 3.1. *MBL2* Gene Polymorphisms

**Figure 2:**
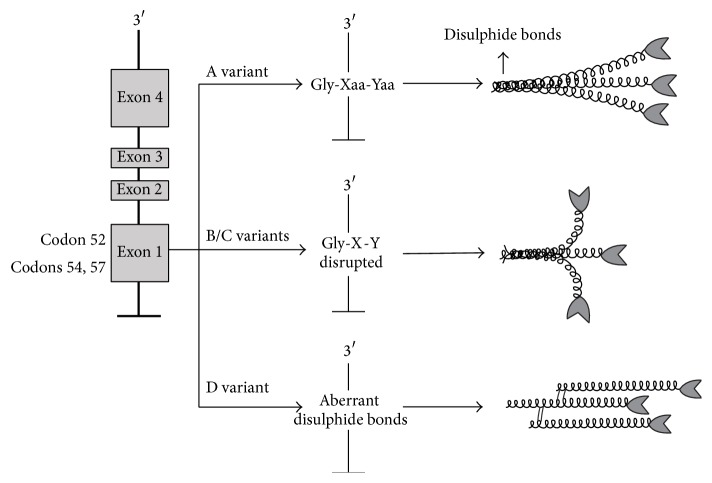
The structural differences due to the variant alleles. Three polymorphisms in the structural gene* MBL2,* at codons 52, 54, and 57, encode for variant alleles referred to as D, B, and C, respectively; the wild-type gene is A. In the wild type, the correct repetition of Glu-Xaa-Yaa permits the association of three identical polypeptide chains generating the structural subunit. This subunit is stabilized through disulphide bonds in the cross-linking region, with a high-order MBL oligomer formation. The mutations in exon 1 generate three amino acid substitutions in the collagen-like region; two of these substitutions disrupt the Gly-X-Y repeats by exchanging a glycine residue with aspartic acid (variant B) or with glutamine (variant C). A third substitutes a cysteine for an arginine (variant D). These amino acid substitutions disrupt the assembly of the MBL molecule, generating a nonfunctional low-order oligomer formation [15, 46].

The presence of variant alleles of the* MBL2* gene encoding three different structural variants of the MBL polypeptide is strongly associated with MBL deficiency. Five single nucleotide polymorphisms (SNPs) in the* MBL2* gene lead to variations in quantity or function of MBL in serum. Two SNPs are localized in the promoter region, at positions −550 (H/L variant) and −221 (X/Y variant), and one is localized in the 5′ untranslated region at position +4 (P/Q variant) [51, 52] (Figure 1). They affect the expression of the MBL2 gene. The haplotypes HY, LY, and LX correlate with high, medium, and low promoter activity, in agreement with the serum measurements [49]. The other three functional SNPs are situated in exon 1, exactly in codon 52 (allele D) [51], in codon 54 (allele B) [2], and in codon 57 (allele C) [53], and result in the disruption of the repeated Gly-Xaa-Yaa structure of the collagenous triple helix by substituting the essential glycine residue with cysteine, aspartic acid, and glutamic acid, respectively [54]. All three variants prevent the assembly of MBL subunits into the basic trimer structure, thereby reducing the amount of MBL protein (Figure 2).

The variant alleles are very frequent in normal, healthy populations, where they are present in 20 to 50% of the individuals, with the highest frequencies found in Africans. The B allele is common in Caucasians, Chinese, and Eskimos with gene frequencies of 0.11 to 0.17%, while the C allele is almost exclusively present in Africans, where it is highly frequent (0.23 to 0.29%). The D allele is present in both Caucasians and Africans, although with a lower frequency (0.05% in both) [14]. All population studies have shown a significant dominant effect of the B, C, and D alleles [51].

MBL serum levels are genetically determined, as described by Sorensen, who estimated the heritability of serum MBL levels and MASP-2 activity in an elegant study on adult twins, underlining the contribution of common genes affecting both traits. The data of this study indicate a strong genetic influence for the serum levels of MBL and for MASP-2 activity, with a significant genetic correlation between the two traits. In fact, twin-twin correlations were higher in monozygotic than in dizygotic twins for both traits, which seem to be influenced, although in part, by the same genes. The genetic correlation may also represent a casual relation between the phenotypes [55].

The genetic variability of both the promoter and the exon domains of MBL gene influences the subsequent stability and serum concentrations of the functionally active protein, leading to defect in opsonization and susceptibility to infections [1].

## 4. MBL: Role in the Activation of Complement in Ischemia/Reperfusion Tissue Injury 

**Figure 3:**
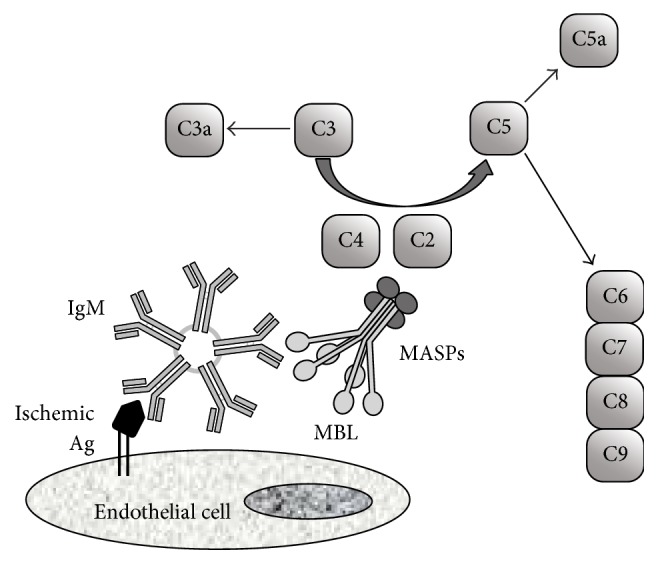
A model illustrates the activation of the lectin pathway by natural IgM in I/R injury. IgM binds to the neoepitope in self-Ag and activates the lectin pathway of complement. The downstream events include the releasing of proinflammatory factors C3a and C5a, deposition of the membrane attack complexes C5–C9, recruitment of inflammatory cells, and leading to direct cell damage [38].

Many studies have shown a determinant role of the complement system in ischemia/reperfusion (I/R) injury in human and animals. Indeed, during the ischemia and in the following reperfusion, the classical pathway has a pivotal role, but the lectin pathways are also involved. Natural circulating IgM (specific to self-antigens) may bind to antigens exposed by ischemia. Antigen interaction initiates the classical pathway, followed by the activation of C1 and downstream components (C4, C3, and C2). The interaction between IgM and ischemic antigen leads to the exposure of binding for the MBL, through the carbohydrate pattern on IgM, and activates the MASPs. The activated MASPSs further cleave relevant substrate activating the lectin pathway (Figure 3). Activated MASP-2 very efficiently cleaves the complement factors C4 and C2 to the fragments C4b and C4a and C2b and C2a, respectively, and C4b and C2b join to form a C3 convertase 2 [30, 56]. MASP-1 can cleave C4b-bound C2, but not C4 [47]. Therefore the lectin pathway activation route is deficient in the absence of MASP-2. MASP-1 can augment MASP-2 functional activity by cleaving C2 and possibly enhancing complement activation by conversion of MASP-2 into the enzymatic active form, but it cannot compensate for the loss of MASP-2 functional activity [57, 58].

This way to the complement activation has been implicated in the pathophysiology of myocardial infarction [59], gastrointestinal ischemia [60], and kidney I/R [61]. A recent study shows the benefits of C1 inhibitor administration in a murine model of cerebral I/R and suggests that MBL is involved in this effect [62].

## 5. MBL: Clinical Significance 

### 5.1. MBL and Susceptibility to Infections

**Figure 4:**
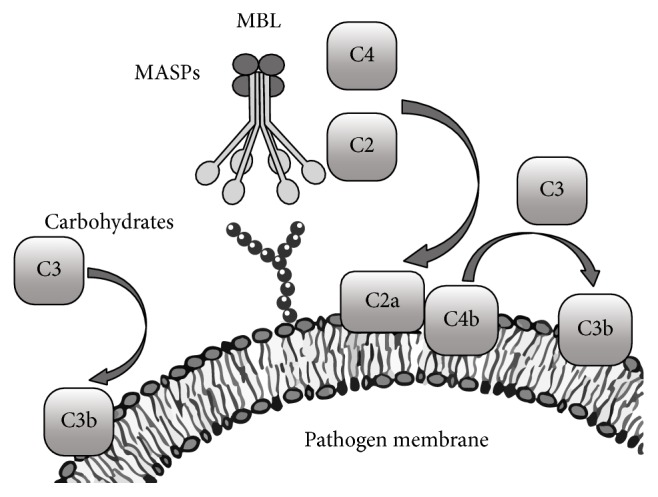
A model illustrates the activation of the lectin pathway by infective agents. MBL recognizes specific carbohydrates such as D-mannose, L-fucose, and N-acetylglucosamine that are represented on the surface of a wide variety of infectious agents. Engagement of ligand by MBL activates MASP2, which then cleaves the C2C4 convertase and results in the cleavage of C3 and the generation of C3b. It has also been proposed that MASP1 can directly cleave C3 [47].

MBL recognizes and binds to sugar moieties on the surface of bacteria, viruses, fungi, and parasites. MBL binding causes these microorganisms to agglutinate and allows phagocytic clearance of pathogens as well as lectin-complement pathway activation, through MBL-associated proteases [63] (Figure 4). Since mounting evidence has supported a crucial role of the MBL in the innate immune response during the last years, several studies focused on the association between MBL expression and/or concentrations in the body fluids and the clinical presentation. Likewise to proteins of the acute phase of inflammation, MBL blood levels increase in response to infections. Healthy adult individuals usually have MBL concentrations above 1000 ng/mL, and these levels seem to be not affected by the age, circadian cycle, and physical exercise. During inflammation, MBL levels increase within the 3-4-fold compared to the baseline level [64]. MBL deficiency in adults has been defined as plasmatic concentrations lower than 500 ng/mL or as an MBL function below 0.2 U/*μ*LC4 deposition [65]. MBL levels may rise under stress to sufficient levels, in individuals who are usually deficient. A positive acute phase response was generally observed in individuals with wild-type MBL2 genes [64].

MBL-deficient adults are characterized by a higher risk, severity, and frequency of infections in a number of clinical settings, although the exact impact of this kind of innate immunodeficiency on the clinical outcome is still poorly understood [66, 67]. Moreover, the risk of developing infections due to low MBL levels seems to be particularly accentuated if associated to other conditions such as cystic fibrosis [68, 69], or after chemotherapy [70–72] and transplantation [73–77].

Nevertheless, despite these results suggesting a protective role of MBL, an excess of MBL activation might be also harmful, due to an unbalanced proinflammatory response leading to additional tissue damage. High MBL activity has been associated with inflammatory autoimmune diseases such as the Systemic Lupus Erythematosus, resulting in organ injury [78]. Furthermore, increased MBL serum concentrations and activity have also been associated with other disorders including transplant rejection [79–84], diabetic nephropathy [85–89], enhanced uptake of mycobacteria [90–93] and* Leishmania* [94–99], and primary biliary cirrhosis [12, 100, 101].

As for the adult population, low MBL levels seem to represent a risk factor also for the development of neonatal infections [6, 7, 102–104]. Particularly low MBL levels have been detected among preterm neonates [64, 105] and a genetically determined MBL deficiency has been described [106, 107], leading to a significant interindividual variability of serum MBL concentrations in the neonatal period.

Low MBL concentrations already in the cord blood were found to correlate with a higher incidence of gram-negative sepsis [4]. Low MBL serum levels on admission to the Neonatal Intensive Care Unit are associated with an increased risk of nosocomial sepsis, independently on gestational age (GA) [6]. Since such low serum MBL concentrations have been reported among septic neonates, a possible role of MBL as biomarker for the early identification of neonates at risk for infection has been suggested [6, 7, 108, 109]. A prospective observational study performed at our institution, which included 365 critically ill neonates, demonstrated that median MBL serum levels were significantly lower among the infected than among the uninfected neonates. Furthermore, low MBL concentrations on admission represented a risk factor for the subsequent development of infection, independently from GA and invasive procedures. Nevertheless, the MBL levels on admission and the peak levels during infection were not associated with death [7].

Schlapbach et al. found a trend towards an increased incidence rate of severe respiratory symptoms in infants with low MBL concentrations, although this association was not as strong as expected [110]. Other authors found that neonates with MASP-2 deficiency had a shorter mean GA, a higher incidence of prematurity, and lower birth weight (BW). Moreover, a trend towards higher MASP-2 concentrations was found among infected neonates [111].

With reference to the correlation between MBL2 genotypes and serum MBL levels on admission in Neonatal Intensive Care Unit, we observed that only 13.8% of our preterm patients carried a genetically deficient* MBL2* haplotype, while 43.1% of babies had deficient MBL levels (<700 ng/mL) on admission to the unit. The finding of a discrepancy between MBL genotypes and serum MBL levels in neonates supports the role of immaturity in causing low MBL levels in neonates. Therefore, in preterm neonates MBL deficiency at birth and in the first month of life seems to be described better by serum concentrations than by MBL genotype [109, 112].

### 5.2. MBL and Adverse Neurological Outcome in Preterm Infants

Robust epidemiological studies, performed in preterm and term-born neonates, suggest a strong association between fetal infection, inflammation (e.g., chorioamnionitis), perinatal brain damage, and neurological disability in term-born infants. Infection and hypoxia-ischemia, despite being very different types of injuries, can individually trigger the fetal inflammatory response, by activation of the fetal immune system, contributing to preterm brain injury, including periventricular white matter injury [9].

In mice or rats with brain insult induced at 5 days of life by ibotenate administration, after systemic injection of IL-1*β*, IL-6, TNF*α*, or IL-9 between first and fifth day of life, a different brain response has been seen, with up to twice the level of brain damage observed in these rodents compared with that observed in nonsensitized animals [113, 114]. Furthermore, in neonates developing later cerebral palsy, an increase of IL-9 plasma level was found without an increase of proinflammatory cytokines. The brain sensitization could be induced by the trigger of neural H1 and H2 receptor, due to release of histamine secondary to the mast cell activation. Also TLR pathway activation (TLR4, TLR3, and adaptor molecule TRIF), leading to cytokines production, seems to be implicated in inflammatory brain sensitization to hypoxia-ischemia insult. The cytokine response due to activation of a common inflammatory pathway could explain the correlation observed between cerebral palsy and the blood cytokine levels of newborn infants born at term [115]. Although a role for neonatal immunity and sepsis has been demonstrated in neonatal encephalopathy, few studies have explored the role of fetal and maternal genetics in predicting the neurological outcome in neonates and whether genetic characteristics of some innate immunity factors may constitute a biomarker of fragility in neonates. In a group of very preterm infants at 24 months of corrected age, we observed that the homozygosity of SNPs of exon 1 of the* MBL2* gene was associated with an adverse neurological outcome. Moreover, all the patients with genotype OO had at least one episode of infection during hospitalization and showed an increased risk for intraventricular hemorrhage (IVH) [70]. So, the effect of* MBL2* SNPs on neurological development could be indirect in these infants, perhaps mediated by the infection, and the brain damage induced by MBL deficiency may be partially independent of complement cascade, less active in such preterm infants than in more mature babies and in adults. Other MBL mediated mechanisms, related to the marked brain immaturity of neonates, may have a role in the genesis of the neurological damage [8].

In a model of traumatic brain injuries in mice, Yager et al. found that MBL deficiency exacerbates acute CA3 (Cornu Ammonis) cells death and cognitive dysfunction, independently of complement activation. This suggests a neuroprotective role for MBL and a functional linkage between innate immunity and neurological outcome after traumatic brain injuries [116]. Yager et al. studied MBL2 genotype in mice and in 135 stroke adult patients (mean age >70 years). At three months of follow-up, they concluded that genetically defined MBL deficiency was associated with a better outcome after acute stroke, without an increased risk of infections in MBL-deficient patients. Moreover, patients with MBL low genotypes disclosed lower serum levels of C3 and C4 than patients with MBL sufficient genotypes [116]. Recently, Cervera et al. confirmed in murine model of middle cerebral artery occlusion the neuroprotective effects of genetic MBL deletion in the acute post-stroke but did not find improvements in either infarct volume or neurological function at 7-day examination [117]. These results are in conflict with each other. If MBL has a protective or harmful role in the physiopathology of the ischemia-reperfusion brain damage is still unclear. According to the studies by Zanetta, we can speculate that MBL may play a protective role in brain development [5]. Mannose rich glycoproteins markedly accumulate during the second and the third postnatal weeks compared with other monosaccharides and are thereafter degraded. They are concentrated at the surface of axons, especially at the surface of parallel fibers (axons of the granule cells, the quantitatively major neuronal cell type in the cerebellum). In premature babies, MBL could promote the contact guidance of neuronal migration, interneuronal recognition, formation of bridges between migrating neurons and radial astrocytes fibers, myelinization, and tightening of the ependymal cell barrier, during the ontogenic development of the brain. A single gene mutation could easily suppress these functions increasing the susceptibility of the brain tissue to various pathogenic insults, as infections and hemorrhages [9].

### 5.3. MBL and Necrotizing Enterocolitis (NEC)

As for adult population, not only MBL deficiency but also MBL hyperproduction seems to have potentially harmful effects. The host immune defence depends on maintaining an appropriate balance between proinflammatory processes and apoptosis. Immaturity of the inflammatory pathways could increase susceptibility to apoptotic activation, upsetting this balance, and result in increased apoptotic tissue damage during bacterial infection. The onset of an excessive and uncontrolled inflammatory response by the neonatal intestine after the exposure to luminal bacteria may trigger the onset of necrotizing enterocolitis. Polymorphisms of the* MBL2* gene associated with high expression of active serum and tissue proteins may predispose preterm neonates to develop NEC and generate the pathophysiology of NEC, which contributes to the disease progression [9].

MBL is expressed by hepatocytes, but Sastry et al. observed low extrahepatic levels of MBL-2 mRNA, predominantly in small intestine [49]. Prencipe et al. detected the expression of MBL protein in the diseased guts of preterm infants with NEC: MBL was strongly expressed in enterocytes, in endothelial cells, and in histiocytes of the small intestine and colon. Moreover, they observed a positive staining for MBL also in enterocytes of intestinal tissues from healthy infants. The −221 promoter MBL-2 variant allele Y, associated with higher serum MBL levels, was shown to be significantly more common in neonates with NEC than in control neonates. Moreover, a significant association of the −221 YY promoter genotype and of the combined exon 1/promoter −221YA/YA genotype, both causing high MBL protein levels, with a higher risk of developing NEC, independently GA, was observed. MBL-2 genotypes related to low MBL levels were shown to be associated with a decreased mortality among neonates with severe NEC, suggesting that MBL levels may affect the outcome of NEC and further supporting the hypothesis of a role of high MBL levels in contributing to intestinal damage [9].

## 6. MBL: Future Perspectives

The observation that low MBL levels represent a risk factor for infection development and severity suggested that the external administration of MBL may be beneficial. Therefore, MBL replacement treatments in critically ill neonates with severe infections are currently discussed, although still far to be applied in clinical practice. However, considering the increased risk of some disorders which have been associated with an uncontrolled production of the MBL (as described above in the text), the potential prophylactic/therapeutic MBL administration should be carefully investigated prior to embarking upon potentially dangerous strategies [12, 118]. Despite the large number of studies investigating the role of MBL, the exact clinical significance of the different MBL haplotypes and, consecutively, the associated serum MBL levels is still poorly understood and needs to be further elucidated, especially in neonates, in which such pathways are not fully developed and functionally mature. In particular, it is still unclear if the response to infection could be blunted or rather exaggerated by the early administration of MBL before the infection development. Population studies have revealed unexpectedly high frequencies of structural MBL gene mutations. It has been suggested that this may reflect a selection advantage for reduced activities of MBL-associated immune mechanisms, such that, for example, individuals with lower levels of MBL might be protected against the complement-mediated damage associated with inflammatory diseases.

The possibility to understand the genetic contribution of specific responses to immunomodulatory agents is a challenge of the current research on infections and inflammatory illness. SNPs of the* MBL2* gene could predict the susceptibility to specific pathogens or complications of infections, allowing us to implement unconventional strategies of prophylaxis and therapy.

In addition, the importance to know the MBL involvement in brain injury, participating in the activation of the inflammatory response, could be important because the induced brain damage due to a first insult makes the developing brain more susceptible to a second insult. Understanding the role of MBL in brain insult and whether the MBL levels could be correlated with neurological outcome could be a possible end point for new study, that is, in newborn with hypoxic/ischemic encephalopathy treated with whole body hypothermia.

## Abbreviations

CRD: Carbohydrate-recognition domain
GA: Gestational age
I/R: Ischemia/reperfusion
IVH: Intraventricular hemorrhage
MASPs: MBL-associated serine proteases
MBL: Mannose-binding lectin
NEC: Necrotizing enterocolitis
SNPs: Single nucleotide polymorphisms.

## References

[1] Kilpatrick D. C. (2002). Mannan-binding lectin: clinical significance and applications. *Biochimica et Biophysica Acta*.

[2] Sumiya M., Super M., Tabona P. (1991). Molecular basis of opsonic defect in immunodeficient children. *The Lancet*.

[3] Scorza M., Liguori R., Elce A., Salvatore F., Castaldo G. (2015). Biological role of mannose binding lectin: from newborns to centenarians. *Clinica Chimica Acta*.

[4] Schlapbach L. J., Mattmann M., Thiel S. (2010). Differential role of the lectin pathway of complement activation in susceptibility to neonatal sepsis. *Clinical Infectious Diseases*.

[5] Zanetta J. P. (2003). Mannose-binding lectins in cerebrum development. *Progress in Molecular and Subcellular Biology*.

[6] De Benedetti F., Auriti C., D'Urbano L. E. (2007). Low serum levels of mannose binding lectin are a risk factor for neonatal sepsis. *Pediatric Research*.

[7] Auriti C., Prencipe G., Inglese R. (2010). Role of mannose-binding lectin in nosocomial sepsis in critically ill neonates. *Human Immunology*.

[8] Auriti C., Prencipe G., Caravale B. (2014). MBL2 gene polymorphisms increase the risk of adverse neurological outcome in preterm infants: a preliminary prospective study. *Pediatric Research*.

[9] Prencipe G., Azzari C., Moriondo M. (2012). Association between mannose-binding lectin gene polymorphisms and necrotizing enterocolitis in preterm infants. *Journal of Pediatric Gastroenterology and Nutrition*.

[10] Turner M. W. (1998). Mannose-binding lectin (MBL) in health and disease. *Immunobiology*.

[11] Kawasaki T. (1999). Structure and biology of mannan-binding protein, MBP, an important component of innate immunity. *Biochimica et Biophysica Acta*.

[12] Kilpatrick D. C. (2003). Introduction to mannan-binding lectin. *Biochemical Society Transactions*.

[13] Klein N. J. (2005). Mannose-binding lectin: do we need it?. *Molecular Immunology*.

[14] Worthley D. L., Bardy P. G., Mullighan C. G. (2005). Mannose-binding lectin: biology and clinical implications. *Internal Medicine Journal*.

[15] Eisen D. P., Minchinton R. M. (2003). Impact of mannose-binding lectin on susceptibility to infectious diseases. *Clinical Infectious Diseases*.

[16] Takahashi K., Ip W. K. E., Michelow I. C., Ezekowitz R. A. B. (2006). The mannose-binding lectin: a prototypic pattern recognition molecule. *Current Opinion in Immunology*.

[17] Mori K., Kawasaki T., Yamashina I. (1984). Subcellular distribution of the mannan-binding protein and its endogenous inhibitors in rat liver. *Archives of Biochemistry and Biophysics*.

[18] Wild J., Robinson D., Winchester B. (1983). Isolation of mannose-binding proteins from human and rat liver. *Biochemical Journal*.

[19] Bouwman L. H., Roos A., Terpstra O. T. (2005). Mannose binding lectin gene polymorphisms confer a major risk for severe infections after liver transplantation. *Gastroenterology*.

[20] Collard C. D., Lekowski R., Jordan J. E., Agah A., Stahl G. L. (1999). Complement activation following oxidative stress. *Molecular Immunology*.

[21] Collard C. D., Vakeva A., Morrissey M. A. (2000). Complement activation after oxidative stress: role of the lectin complement pathway. *American Journal of Pathology*.

[22] Malhotra R., Willis A. C., Lopez Bernal A., Thiel S., Sim R. B. (1994). Mannan-binding protein levels in human amniotic fluid during gestation and its interaction with collectin receptor from amnion cells. *Immunology*.

[23] Garred P., Brygge K., Sorensen C. H. (1993). Mannan binding protein levels in plasma and upper airway secretions and frequency of genotypes in children with recurrence of otitis media. *Clinical & Experimental Immunology*.

[24] Bohlson S. S., O'Conner S. D., Hulsebus H. J., Ho M.-M., Fraser D. A. (2014). Complement, C1Q, and C1q-related molecules regulate macrophage polarization. *Frontiers in Immunology*.

[25] Fraser D. A., Tenner A. J. (2008). Directing an appropriate immune response: the role of defense collagens and other soluble pattern recognition molecules. *Current Drug Targets*.

[26] Nadesalingam J., Dodds A. W., Reid K. B. M., Palaniyar N. (2005). Mannose-binding lectin recognizes peptidoglycan via the *N*-acetyl glucosamine moiety, and inhibits ligand-induced proinflammatory effect and promotes chemokine production by macrophages. *The Journal of Immunology*.

[27] Casanova J.-L., Abel L. (2004). Human mannose-binding lectin in immunity: friend, foe, or both?. *Journal of Experimental Medicine*.

[28] Grumach A. S., Ceccon M. E., Rutz R., Fertig A., Kirschfink M. (2014). Complement profile in neonates of different gestational ages. *Scandinavian Journal of Immunology*.

[29] Neth O., Jack D. L., Johnson M., Klein N. J., Turner M. W. (2002). Enhancement of complement activation and opsonophagocytosis by complexes of mannose-binding lectin with mannose-binding lectin-associated serine protease after binding to Staphylococcus aureus. *Journal of Immunology*.

[30] Thiel S., Vorup-Jensen T., Stover C. M. (1997). A second serine protease associated with mannan-binding lectin that activates complement. *Nature*.

[31] Matsushita M., Fujita T. (1992). Activation of the classical complement pathway by mannose-binding protein in association with a novel C1s-like serine protease. *The Journal of Experimental Medicine*.

[32] Matsushita M., Fujita T. (1995). Cleavage of the third component of complement (C3) by mannose-binding protein-associated serine protease (MASP) with subsequent complement activation. *Immunobiology*.

[33] Dahl M. R., Thiel S., Matsushita M. (2001). MASP-3 and its association with distinct complexes of the mannan-binding lectin complement activation pathway. *Immunity*.

[34] Degn S. E., Jensen L., Hansen A. G. (2012). Mannan-binding lectin-associated serine protease (MASP)-1 is crucial for lectin pathway activation in human serum whereas neither MASP-1 nor MASP-3 is required for alternative pathway function. *Journal of Immunology*.

[35] Liu Y., Endo Y., Iwaki D. (2005). Human M-ficolin is a secretory protein that activates the lectin complement pathway. *Journal of Immunology*.

[36] Matsushita M., Endo Y., Fujita T. (2000). Cutting edge: complement-activating complex of ficolin and mannose-binding lectin-associated serine protease. *Journal of Immunology*.

[37] Matsushita M., Kuraya M., Hamasaki N., Tsujimura M., Shiraki H., Fujita T. (2002). Activation of the lectin complement pathway by H-ficolin (Hakata antigen). *The Journal of Immunology*.

[38] Zhang M., Takahashi K., Alicot E. M. (2006). Activation of the lectin pathway by natural IgM in a model of ischemia/reperfusion injury. *The Journal of Immunology*.

[39] Kuraya M., Matsushita M., Endo Y., Thiel S., Fujita T. (2003). Expression of H-ficolin/Hakata antigen, mannose-binding lectin-associated serine protease (MASP)-1 and MASP-3 by human glioma cell line T98G. *International Immunology*.

[40] Morio H., Kurata H., Katsuyama R., Oka S., Kozutsumi Y., Kawasaki T. (1997). Renal expression of serum-type mannan-binding protein in rat. *European Journal of Biochemistry*.

[41] Vorup-Jensen T., Sørensen E. S., Jensen U. B. (2001). Recombinant expression of human mannan-binding lectin. *International Immunopharmacology*.

[42] Wagner S., Lynch N. J., Walter W., Schwaeble W. J., Loos M. (2003). Differential expression of the murine mannose-binding lectins A and C in lymphoid and nonlymphoid organs and tissues. *The Journal of Immunology*.

[43] Grasso D. L., Segat L., Zocconi E., Radillo O., Trevisiol C., Crovella S. (2009). MBL expression in patients with recurrent tonsillitis. *International Journal of Pediatric Otorhinolaryngology*.

[44] Seyfarth J., Garred P., Madsen H. O. (2006). Extra-hepatic transcription of the human mannose-binding lectin gene (mbl2) and the MBL-associated serine protease 1-3 genes. *Molecular Immunology*.

[45] Babula O., Lazdane G., Kroica J., Ledger W. J., Witkin S. S. (2003). Relation between recurrent vulvovaginal candidiasis, vaginal concentrations of mannose-binding lectin, and a mannose-binding lectin gene polymorphism in latvian women. *Clinical Infectious Diseases*.

[46] Petersen S. V., Thiel S., Jensenius J. C. (2001). The mannan-binding lectin pathway of complement activation: biology and disease association. *Molecular Immunology*.

[47] Ambrus G., Gál P., Kojima M. (2003). Natural substrates and inhibitors of mannan-binding lectin-associated serine protease-1 and -2: a study on recombinant catalytic fragments. *Journal of Immunology*.

[48] Taylor M. E., Brickell P. M., Craig R. K., Summerfield J. A. (1989). Structure and evolutionary origin of the gene encoding a human serum mannose-binding protein. *Biochemical Journal*.

[49] Sastry K., Herman G. A., Day L. (1989). The human mannose-binding protein gene. Exon structure reveals its evolutionary relationship to a human pulmonary surfactant gene and localization to chromosome 10. *Journal of Experimental Medicine*.

[50] Garred P., Larsen F., Madsen H. O., Koch C. (2003). Mannose-binding lectin deficiency—revisited. *Molecular Immunology*.

[51] Madsen H. O., Garred P., Thiel S. (1995). Interplay between promoter and structural gene variants control basal serum level of mannan-binding protein. *The Journal of Immunology*.

[52] Madsen H. O., Garred P., Kurtzhals J. A. L. (1994). A new frequent allele is the missing link in the structural polymorphism of the human mannan-binding protein. *Immunogenetics*.

[53] Lipscombe R. J., Sumiya M., Hill A. V. S. (1992). High frequencies in African and non-African populations of independent mutations in the mannose binding protein gene. *Human Molecular Genetics*.

[54] Steffensen R., Thiel S., Varming K., Jersild C., Jensenius J. C. (2000). Detection of structural gene mutations and promoter polymorphisms in the mannan-binding lectin (MBL) gene by polymerase chain reaction with sequence-specific primers. *Journal of Immunological Methods*.

[55] Sorensen G. L., Petersen I., Thiel S. (2007). Genetic influences on Mannan-binding lectin (MBL) and Mannan-binding lectin associated serine protease-2 (MASP-2) activity. *Genetic Epidemiology*.

[56] Matsushita M., Thiel S., Jensenius J. C., Terai I., Fujita T. (2000). Proteolytic activities of two types of mannose-binding lectin-associated serine protease. *The Journal of Immunology*.

[57] Farrar C. A., Asgari E., Schwaeble W. J., Sacks S. H. (2012). Which pathways trigger the role of complement in ischaemia/reperfusion injury?. *Frontiers in Immunology*.

[58] Zhang M., Takahashi K., Alicot E. M. (2006). Activation of the lectin pathway by natural IgM in a model of ischemia/reperfusion injury. *Journal of Immunology*.

[59] Jordan J. E., Montalto M. C., Stahl G. L. (2001). Inhibition of mannose-binding lectin reduces postischemic myocardial reperfusion injury. *Circulation*.

[60] Hart M. L., Ceonzo K. A., Shaffer L. A. (2005). Gastrointestinal ischemia-reperfusion injury is lectin complement pathway dependent without involving C1q. *Journal of Immunology*.

[61] De Vries B., Walter S. J., Peutz-Kootstra C. J., Wolfs T. G. A. M., Van Heurn L. W. E., Buurman W. A. (2004). The mannose-binding lectin-pathway is involved in complement activation in the course of renal ischemia-reperfusion injury. *American Journal of Pathology*.

[62] Gesuete R., Storini C., Fantin A. (2009). Recombinant C1 inhibitor in brain ischemic injury. *Annals of Neurology*.

[63] Lau Y. L., Chan S. Y., Turner M. W., Fong J., Karlberg J. (1995). Mannose-binding protein in preterm infants: developmental profile and clinical significance. *Clinical and Experimental Immunology*.

[64] Dean M. M., Minchinton R. M., Heatley S., Eisen D. P. (2005). Mannose binding lectin acute phase activity in patients with severe infection. *Journal of Clinical Immunology*.

[65] Eisen D. P., Dean M. M., Boermeester M. A. (2008). Low serum mannose-binding lectin level increases the risk of death due to pneumococcal infection. *Clinical Infectious Diseases*.

[66] Eisen D. P., Dean M. M., Thomas P. (2006). Low mannose-binding lectin function is associated with sepsis in adult patients. *FEMS Immunology and Medical Microbiology*.

[67] De Pascale G., Cutuli S. L., Pennisi M. A., Antonelli M. (2013). The role of mannose-binding lectin in severe sepsis and septic shock. *Mediators of Inflammation*.

[68] Gravina L. P., Crespo C., Giugno H. (2015). Mannose-binding lectin gene as a modifier of the cystic fibrosis phenotype in Argentinean pediatric patients. *Journal of Cystic Fibrosis*.

[69] Garred P., Pressler T., Madsen H. O. (1999). Association of mannose-binding lectin gene heterogeneity with severity of lung disease and survival in cystic fibrosis. *Journal of Clinical Investigation*.

[70] Neth O., Hann I., Turner M. W., Klein N. J. (2001). Deficiency of mannose-binding lectin and burden of infection in children with malignancy: a prospective study. *The Lancet*.

[71] Peterslund N. A., Koch C., Jensenius J. C., Thiel S. (2001). Association between deficiency of mannose-binding lectin and severe infections after chemotherapy. *Lancet*.

[72] Chalmers J. D., Fleming G. B., Hill A. T., Kilpatrick D. C. (2011). Impact of mannose-binding lectin insufficiency on the course of cystic fibrosis: a review and meta-analysis. *Glycobiology*.

[73] Moreto A., Fariñas-Alvarez C., Puente M. (2014). Mannose-binding lectin gene variants and infections in patients receiving autologous stem cell transplantation. *BMC Immunology*.

[74] Wan Q.-Q., Ye Q.-F., Zhou J.-D. (2013). Mannose-binding lectin 2 and ficolin-2 gene polymorphisms influence the susceptibility to bloodstream infections in kidney transplant recipients. *Transplantation Proceedings*.

[75] Stevenson H. L., Amador A., McCue J. (2013). Mannose binding lectin (mbl2) haplotype frequencies in solid organ transplant patients and correlation with MBL protein levels—evaluation of complement-mediated effector pathway deficiency. *Transplant Immunology*.

[76] Damman J., Seelen M. A. (2013). Mannan binding lectin: a two-faced regulator of renal allograft injury?. *Kidney International*.

[77] Budd S. J., Aris R. M., Medaiyese A. A., Tilley S. L., Neuringer I. P. (2012). Increased plasma mannose binding lectin levels are associated with bronchiolitis obliterans after lung transplantation. *Respiratory Research*.

[78] Pradhan V., Surve P., Ghosh K. (2010). Mannose binding lectin (MBL) in autoimmunity and its role in systemic lupus erythematosus (SLE). *Journal of Association of Physicians of India*.

[79] Golshayan D., Wójtowicz A., Bibert S. (2016). Polymorphisms in the lectin pathway of complement activation influence the incidence of acute rejection and graft outcome after kidney transplantation. *Kidney International*.

[80] Eurich D., Boas-Knoop S., Yahyazadeh A. (2012). Role of mannose-binding lectin-2 polymorphism in the development of acute cellular rejection after transplantation for hepatitis C virus-induced liver disease. *Transplant Infectious Disease*.

[81] Ibernon M., Moreso F., Serón D. (2011). Subclinical rejection in renal transplants is associated with low serum mannose-binding lectin levels. *Kidney International Supplements*.

[82] Carroll K. E., Dean M. M., Heatley S. L. (2011). High levels of mannose-binding lectin are associated with poor outcomes after lung transplantation. *Transplantation*.

[83] Fildes J. E., Shaw S. M., Walker A. H. (2008). Mannose-binding lectin deficiency offers protection from acute graft rejection after heart transplantation. *Journal of Heart and Lung Transplantation*.

[84] Fiane A. E., Ueland T., Simonsen S. (2005). Low mannose-binding lectin and increased complement activation correlate to allograft vasculopathy, ischaemia, and rejection after human heart transplantation. *European Heart Journal*.

[85] Zhao S.-Q., Hu Z. (2016). Mannose-binding lectin and diabetic nephropathy in type 1 diabetes. *Journal of Clinical Laboratory Analysis*.

[86] Guan L.-Z., Tong Q., Xu J. (2015). Elevated serum levels of mannose-binding lectin and diabetic nephropathy in type 2 diabetes. *PLoS ONE*.

[87] Zhang N., Zhuang M., Ma A. (2013). Association of levels of Mannose-binding lectin and the *MBL2*gene with type 2 diabetes and diabetic nephropathy. *PLoS ONE*.

[88] Hansen T. K., Forsblom C., Saraheimo M. (2010). Association between mannose-binding lectin, high-sensitivity C-reactive protein and the progression of diabetic nephropathy in type 1 diabetes. *Diabetologia*.

[89] Saraheimo M., Forsblom C., Hansen T. K. (2005). Increased levels of mannan-binding lectin in type 1 diabetic patients with incipient and overt nephropathy. *Diabetologia*.

[90] Świerzko A. S., Bartłomiejczyk M. A., Brzostek A. (2016). Mycobacterial antigen 85 complex (Ag85) as a target for ficolins and mannose-binding lectin. *International Journal of Medical Microbiology*.

[91] Bartlomiejczyk M. A., Swierzko A. S., Brzostek A., Dziadek J., Cedzynski M. (2014). Interaction of lectin pathway of complement-activating pattern recognition molecules with *Mycobacteria*. *Clinical and Experimental Immunology*.

[92] Singla N., Gupta D., Joshi A., Batra N., Singh J., Birbian N. (2012). Association of mannose-binding lectin gene polymorphism with tuberculosis susceptibility and sputum conversion time. *International Journal of Immunogenetics*.

[93] Søborg C., Madsen H. O., Andersen Å. B., Lillebaek T., Kok-Jensen A., Garred P. (2003). Mannose-binding lectin polymorphisms in clinical tuberculosis. *Journal of Infectious Diseases*.

[94] Mishra A., Antony J. S., Gai P. (2015). Mannose-binding Lectin (MBL) as a susceptible host factor influencing Indian Visceral Leishmaniasis. *Parasitology International*.

[95] De Araujo F. J., Mesquita T. G., Da Silva L. D. O. (2015). Functional variations in *MBL2*gene are associated with cutaneous leishmaniasis in the Amazonas state of Brazil. *Genes and Immunity*.

[96] Asgharzadeh M., Mazloumi A., Kafil H. S., Ghazanchaei A. (2007). Mannose-binding lectin gene and promoter polymorphism in visceral leishmaniasis caused by Leishmania infantum. *Pakistan Journal of Biological Sciences*.

[97] Alonso D. P., Ferreira A. F. B., Ribolla P. E. M. (2007). Genotypes of the mannan-binding lectin gene and susceptibility to visceral leishmaniasis and clinical complications. *Journal of Infectious Diseases*.

[98] Ambrosio A. R., De Messias-Reason I. J. T. (2005). Leishmania (*Viannia*) *braziliensis*: interaction of mannose-binding lectin with surface glycoconjugates and complement activation. An antibody-independent defence mechanism. *Parasite Immunology*.

[99] De Miranda Santos I. K. F., Costa C. H. N., Krieger H. (2001). Mannan-binding lectin enhances susceptibility to visceral leishmaniasis. *Infection and Immunity*.

[100] Matsushita M., Miyakawa H., Tanaka A. (2001). Single nucleotide polymorphisms of the mannose-binding lectin are associated with susceptibility to primary biliary cirrhosis. *Journal of Autoimmunity*.

[101] Bouwman L. H., Roep B. O., Roos A. (2006). Mannose-binding lectin: clinical implications for infection, transplantation, and autoimmunity. *Human Immunology*.

[102] Fidler K. J., Wilson P., Davies J. C., Turner M. W., Peters M. J., Klein N. J. (2004). Increased incidence and severity of the systemic inflammatory response syndrome in patients deficient in mannose-binding lectin. *Intensive Care Medicine*.

[103] Gordon A. C., Waheed U., Hansen T. K. (2006). Mannose-binding lectin polymorphisms in severe sepsis: relationship to levels, incidence, and outcome. *Shock*.

[104] Stephens R. C. M., Fidler K., Wilson P. (2006). Endotoxin immunity and the development of the systemic inflammatory response syndrome in critically ill children. *Intensive Care Medicine*.

[105] Dzwonek A. B., Neth O. W., Thiébaut R. (2008). The role of mannose-binding lectin in susceptibility to infection in preterm neonates. *Pediatric Research*.

[106] Koroglu O. A., Onay H., Erdemir G. (2010). Mannose-binding lectin gene polymorphism and early neonatal outcome in preterm infants. *Neonatology*.

[107] Israëls J., Frakking F. N. J., Kremer L. C. M., Offringa M., Kuijpers T. W., Van De Wetering M. D. (2010). Mannose-binding lectin and infection risk in newborns: a systematic review. *Archives of Disease in Childhood: Fetal and Neonatal Edition*.

[108] Wahab Mohamed W. A., Saeed M. A. (2012). Mannose-binding lectin serum levels in neonatal sepsis and septic shock. *Journal of Maternal-Fetal and Neonatal Medicine*.

[109] St Swierzko A., Szala A., Cedzynski M. (2009). Mannan-binding lectin genotypes and genotype-phenotype relationships in a large cohort of Polish neonates. *Human Immunology*.

[110] Schlapbach L. J., Latzin P., Regamey N. (2009). Mannose-binding lectin cord blood levels and respiratory symptoms during infancy: a prospective birth cohort study. *Pediatric Allergy and Immunology*.

[111] Swierzko A. S., Cedzynski M., Domzalska-Popadiuk I. (2009). Mannan-binding lectin-associated serine protease-2 (MASP-2) in a large cohort of neonates and its clinical associations. *Molecular Immunology*.

[112] Oudshoorn A.-M. J., van den Dungen F. A. M., Bach K. P. (2008). Mannose-binding lectin in term newborns and their mothers: genotypic and phenotypic relationship. *Human Immunology*.

[113] Dommergues M.-A., Patkai J., Renauld J.-C., Evrard P., Gressens P. (2000). Proinflammatory cytokines and interleukin-9 exacerbate excitotoxic lesions of the newborn murine neopallium. *Annals of Neurology*.

[114] Patkai J., Mesples B., Dommergues M.-A. (2001). Deleterious effects of IL-9 -activated mast cells and neuroprotection by antihistamine drugs in the developing mouse brain. *Pediatric Research*.

[115] Nelson K. B., Dambrosia J. M., Grether J. K., Phillips T. M. (1998). Neonatal cytokines and coagulation factors in children with cerebral palsy. *Annals of Neurology*.

[116] Yager P. H., You Z., Qin T. (2008). Mannose binding lectin gene deficiency increases susceptibility to traumatic brain injury in mice. *Journal of Cerebral Blood Flow and Metabolism*.

[117] Cervera A., Planas A. M., Justicia C. (2010). Genetically-defined deficiency of mannose-binding lectin is associated with protection after experimental stroke in mice and outcome in human stroke. *PLoS ONE*.

[118] Kilpatrick D. C. (2003). Consensus statement on the future of mannan-binding lectin (MBL)-replacement therapy. *Biochemical Society Transactions*.

